# Feasibility and acceptability of integrating hepatitis B care into routine HIV services: a qualitative study among health care providers and patients in West Nile region, Uganda

**DOI:** 10.1186/s12913-022-08924-0

**Published:** 2023-01-20

**Authors:** Joan Nankya Mutyoba, Claude Wandera, David Ejalu, Emmanuel Seremba, Rachel Beyagira, Jacinto Amandua, Kaggwa Mugagga, Andrew Kambugu, Alex Muganzi, Philippa Easterbrook, Ponsiano Ocama

**Affiliations:** 1 Department of Epidemiology and Biostatistics, School of Public Health, Makerere University College of Health Sciences, P.O. Box 7072, Kampala, Uganda; 2 Infectious Diseases Institute, Makerere University College of Health Sciences, Kampala, Uganda; 3 Department of Medicine, School of Medicine, Makerere University College of Health Sciences, Kampala, Uganda; 4 Program on viral Hepatitis, Ministry of Health, Kampala, Uganda; 5 WHO, Kampala, Uganda

**Keywords:** Feasibility, HIV, Hepatitis B, Integration, Uganda

## Abstract

**Background:**

Despite facing a dual burden of HBV and HIV, Africa lacks experience in offering integrated care for HIV and HBV. To contextualize individual and group-level feasibility and acceptability of an integrated HIV/HBV care model, we explored perspectives of health care providers and care recipients on feasibility and acceptability of integration.

**Methods:**

In two regional hospitals of West Nile region, we performed a demonstration project to assess feasibility and acceptability of merging the care of HBV-monoinfected patients with existing HIV care system. Using interviews with health care providers as key informants, and 6 focus groups discussions with 3 groups of patients, we explored feasibility [(i)whether integration is perceived to fit within the existing healthcare infrastructure, (ii) perceived ease of implementation of HIV/HBV integrated care, and (iii) perceived sustainability of integration] and acceptability [whether the HIV/HBV care model is perceived as (i) suitable, (ii) satisfying and attractive (iii) there is perceived demand, need and intention to recommend its use]. We audio-recorded the interviews and data was analysed using framework analysis.

**Results:**

The following themes emerged from the data (i) integrating HBV into HIV care is perceived to be feasible, fit and beneficial, after making requisite adjustments (ii) integration is acceptable due to the need for both free treatment and anticipated collaboration between HIV and HBV clients in terms of peer-support (iii) there are concerns about the likely rise in stigma and the lack of community awareness about integrated care.

**Conclusion:**

The integrated HIV/HBV care model is feasible and acceptable among both providers and recipients. Necessary adjustments to the existing care system, including training, for community sensitization on the reasons and significance of integration are required.

**Supplementary Information:**

The online version contains supplementary material available at 10.1186/s12913-022-08924-0.

## Introduction

Africa currently faces a dual burden of chronic hepatitis B virus (HBV) and HIV. Chronic HBV affects an estimated 5–8% of the continent’s population [1–3]. The HBV prevalence is highest in countries within West Africa, but countries elsewhere, including Uganda still bear a significant load of this disease. For Uganda, about 4.1% of the population is estimated to have chronic HBV infection (defined as presence of hepatitis B surface antigen), and regionally-disaggregated data reveals a higher prevalence (4.6%) of HBV infection in the Northern region of the Country [4]. On the other hand, prevalence of HIV in Uganda is 6.2% in the population aged between 15 and 64 years. The prevalence of HIV in West Nile region is 3.1%. More recent data from the northern region found a prevalence of HIV/HBV co-infection of about 8% [5]. Uganda, like most sub-Saharan Africa (SSA) has a functional infrastructure and service delivery system for HIV care, but a similar system is lacking for HBV. Currently, both HIV and HBV are managed in separate clinics, with separate staff teams and they all receive antiretroviral treatment. Unlike the HIV clinic, patients in the HBV clinic do not receive routine counseling and education, have very limited resources for laboratory investigations, and have higher loss to follow-up, partly due to lack of a community support system to ensure they remain in continuous care. HBV-infected persons are not being tested for HIV, and patients in the HIV clinics do not undergo routine hepatitis testing, or education. HIV services, including HIV testing, have been integrated in routine services at primary care level in most African countries. In addition, over the past decade and due to significant funding, high quality HIV care systems have been established across high-burden HIV regions of Africa. However, similar care services for HBV are lacking on most high HBV burden settings. Despite the availability of now comprehensive guidelines on HBV care and management for low-income settings [6], challenges persist in actual implementation. This is mostly due to lack of resources for adequate management of hepatitis B patients. Moreover, HBV patients in care need to be monitored for HIV status. The two infections have similar modes of transmission and there is a clear overlap in their diagnosis, care and management which strengthens the possibility of integrating their care. Care integration would also enable optimal use of the limited resources including health care personnel, clinic space and laboratory support system to offer continuous care to hepatitis B patients. Care integration may however, bring additional challenges including drug stock-outs and increased workload on staff serving in the HIV Clinics.

A key obstacle to effective implementation of HBV/HIV care integration is that feasibility studies on how to implement this within a context of limited resources, and its acceptability among key stakeholders including care providers and patients are scarce. We therefore performed a demonstration project to examine how feasible, acceptable it is, to integrate HBV care into the existing HIV care delivery system, using available resources.

This qualitative study was theoretically underpinned on the theoretical framework for acceptability (TFA), a framework that defines acceptability as “ *a multi-faceted construct that reflects the extent to which people delivering or receiving a healthcare intervention consider it to be appropriate, based on anticipated or experienced cognitive and emotional responses to the intervention* ” [7]. Acceptability of health care interventions is thought to improve not only patient’s full engagement with and navigation of the necessary care processes, but also increases adherence to integrated care [8]. This is mainly because it involves not just the patient’s evaluation of benefits and costs, but also a reflection of personal needs and preferences and the extent to which these are met by the attributes of the health care intervention. Therefore a deep understanding of acceptability would improve overall feasibility of intervention uptake by informing ways to tailor interventions to the needs of end-users [9] and thereby raising end-user satisfaction.

To gain an understanding of feasibility and acceptability from the perspective of patients and care providers, we qualitatively explored views relating to how feasible it is to integrate and how acceptable this integrated HIV/HBV care model is, as well as the likely influential factors. Although some countries have mentioned the possibility of exploring HBV/HIV care integration, [10–12] fewer have actually taken steps to implement integration. This exposes a void in information on the experience and practice of providing integrated care that is holistic for both HIV- and HBV-infected persons.

Successful planning of integration, however needs input from end-users of the service in order to effectively inform program feasibility as well as acceptability. This study therefore, was a qualitative inquiry into both patients and providers’ views on feasibility and acceptability of integrating HBV care into routine HIV services. The objective was to explore perceptions on degree of, and the extent to which, the HBV/HIV integrated pathway is judged as suitable, satisfying, or attractive to program deliverers and program recipients.

## Methods

### Study setting

The study was embedded in a wider demonstration project that aimed to integrate HBV care into routine HIV care delivery system, known as the “2for1” demonstration project. Aside from the feasibility and acceptability component being reported here, the project also had components of training healthcare workers on care and management of HBV infection whether as mono- or co-infection, developing pathways for HBV and HIV standalone clinics compared to integrated pathway and costing the pathways. The rest of the components are being written separately. It was implemented in two public health facilities, Arua regional referral hospital and Koboko district hospital, both located in North-Western Uganda. This region has a significant refugee population [13] and a high burden of HBV [5, 14]. Arua regional referral hospital is a higher level facility with a high patient volume and a 323 bed-capacity. It serves a population of 782,077 including districts of West Nile and Northern Uganda [15]. Koboko hospital is a lower level facility which serves a population of 129,148 in a region that shares a border with both South Sudan and the Democratic Republic of Congo.

### Study design and sample selection

The study utilized focus group discussions (FGD) and key-informant interviews (KII). In each facility, three groups of participants were purposively enrolled for FGDs;(i) a group of HIV-infected patients; (ii) a group of HBV-infected patients; (iii) a group of patients with HIV and HBV co-infection. This design yielded a set of 6 groups of participants with a mixed background regarding age, sex, ethnicity, socioeconomic status, religious, cultural and health beliefs. Yet, relative group homogeneity arising from a common chronic infectious illness would allow free interactions between participants and free expression of personal views relevant to the discussion [16]. Key informants were consecutively selected and these included different cadres of health care providers. Participants were physically approached. In Arua, we interviewed 11 health care workers (HCWs), five of whom were female, while in Koboko we interviewed 9 HCWs, five of whom were female. The HIV-focus group in Arua had 8 participants, 4 of whom were female, in Koboko it had 10 participants, 4 of whom were female. The HBV-focus group in Arua had 10 participants, 5 of whom were female, in Koboko it had 7 participants, 3 of whom were female. The HIV/HBV-focus group in Arua had 11 participants, 5 of whom were female, in Koboko it had 6 participants, 2 of whom were female. Study participants were purposively selected from those who had prior experience journeying through either the HIV or the HBV care processes, or both for more than a year. Patients had to be attending the clinics at the study sites, while health care providers had to be working in either the hepatitis clinic or the HIV clinic. The study was introduced to patients who had come for services during the health education session, and those willing to join were consecutively selected until the required number was reached, per focus group. Health care workers who were most senior and had worked in the HIV or HBV clinics longest were selected, because they had experience with patient care processes. Focus group discussions were convened after patients had received care, because this was their preference.

### Study tools and data collection

Data was collected prior to HIV/HBV integration and this was done at the respective health facilities. Semi-structured interview guides were used to guide both the KII and the FGD. Both the study objectives and theoretically-informed constructs of health intervention feasibility and acceptability [7, 17] guided the design of study tools. Each tool had a total of 15 open-ended questions distributed across two sections; perceived feasibility and acceptability. For perceived feasibility to integrate HBV into HIV care model we explored whether (i) integration is perceived to fit within the existing healthcare infrastructure, (ii) perceived ease of implementation of HIV/HBV integrated care, and (iii) perceived sustainability of integration. For acceptability of the integrated HIV/HBV care model, we explored whether the HIV/HBV care model is perceived as (i) suitable, (ii) satisfying and attractive (iii) there is perceived demand, need and intention to recommend its use. All study tools were translated and back translated into *Lugbara* and *Akakwa* for Arua and Koboko regions respectively. They were also piloted among attendees of outpatient clinics of the hospitals. A private room within each hospital setting was provided, where interviews with key informants and focus group discussions were conducted. Both the KIIs and FGDs were facilitated by two trained research assistants with expertise in qualitative interviewing. One facilitator moderated the discussion while the other managed the audio-recording. Both took notes during the sessions. Probing techniques were used to allow participants share complete information on issues that emerged. Data was collected until saturation was achieved. Back-up notes were taken during each interview or FGD and updated into descriptive narratives soon after the sessions. Interviews and focus group discussions took place in a private setting to ensure confidentiality. Individual interviews lasted about 50 minutes, while focus group discussions lasted about 90 minutes each.

### Data analysis

Data from the audio recordings were translated into English and precisely transcribed by research assistants and one of the investigators. Care was taken to maintain meaning during transcription. We conceptually based our analysis on the framework analysis [18, 19]. Three investigators read the interview text several times to gain immersion into the data. Then, parts of text were condensed into meaning units and similar meaning units were compiled and given a code after discussion and agreement among the investigators. Coding process used both inductive method that generated emerging themes and deductive approaches, with pre-selected themes. Codes were then compared and sorted into categories. Interpretation of categories for latent meaning then led to emerging themes and sub-themes, which were presented with corresponding supporting quotes. The coding matrix has been provided as additional file 1.

### Reflexivity aspects

Data collection and analysis was led by one female and 2 male researchers with relevant experience in qualitative research in public health. Authors JNM (MD, MS, PhD), CW (MPH) and DE (MHSR) performed the KIIs and facilitated the FGDs. They all had training in qualitative research and analysis methods and were study investigators. Interviewers greeted and introduced themselves to participants prior to the interview. Participants were not made aware of interviewers’ particular interests in the study, including whether or not they preferred HBV care integration. Individual researchers may however, have had undisclosed assumptions regarding when or how best to integrate HIV/HBV care. The COREQ guidelines were used and the checklist is provided as additional file 2.

### Ethical aspects

The study received approval from Makerere University School of Medicine Research Ethics committee (SOMREC REC REF 2018 − 185) and Uganda National Council for Science and Technology (UNCST SS 4986) and carried out following the Declaration of Helsinki protocol. All study participants were provided with complete information about the study, and taken through a consenting process prior to study participation. Data was collected in a quiet private environment and information provided by participants was treated with confidentiality.

## Results

A total of 20 health care workers participated in key-informant interviews (Table 1). They included 3 physicians, 2 medical officers, 3 physician assistants, 2 pharmacy technicians, 4 nurses, 2 data officers and 4 laboratory technicians. There were 52 patient participants in focus group discussions. None of the participants that were approached to participate in the study refused participation. No participant dropped out of the study.

**Table 1:** Showing distribution of focus group discussion and key informant interview participants in Arua and Koboko hospitals in West Nile region, Uganda

	**Koboko district hospital**	**Arua regional referral hospital**
**Focus-group discussions-(patient-groups)**		
HIV-infected group	10	8
HBV-infected group	7	10
HIV/HBV co-infected group	6	11
**Key informant interview participants** (**HCWs**)^a^	9	11

^a^*HCWs* Health Care Workers

Findings from the study revealed that (i) integrating HBV into HIV care is feasible, fit and appropriate, after making requisite adjustments (ii) integration acceptable due to the need for both free treatment and anticipated collaboration with HBV clients to strengthen peer support (iii) there were concerns regarding likely rise in stigma (iv) there is a need for community sensitization on the reasons and significance of integration.

## Meaning and perceived feasibility of HIV/HBV care integration

When asked to describe what they understood by the term “integration of care”, all three FGDs had comparable interpretation of “HIV/HBV care integration”. They mostly perceived it as providing care to HIV- and HBV-patients concurrently, and they noted that both patient groups take similar medications. The FGD of hepatitis clients in Arua explained it as “*… bringing the two people together because they are all taking the same drugs*” and from the FGD of HIV clients, “*Integration means trying to see how they can bring people of HIV and HBV together for their treatment in the same place”*. The FGD of HIV/HBV co-infection understood integration as a way to provide better patient management. Their meaning of integration bore a common thread of purposing to improve patient care services.

Most participants viewed integration as feasible, suitable and beneficial. They noted that the HIV clinic was well organized with all requirements needed to support the HBV clients too. Most FGD-groups however, weighed perspectives of feasibility through the lens of how accessible and easy the integrated services might be, including convenience in navigating care.

### Integration- an opportunity to leverage the existing HIV care system for HBV care

*“Combining [the care of HBV with HIV] is a better alternative as per now because we know we are going to use the same human resource; if their capacity is built to handle both [infections] at the same time it will be an advantage where we might not lack staffing. Integration is very suitable for our system because the two [infections] are almost similar“* Nurse 02, KII, Koboko

In keeping with the view that integration was appropriate, both HCWs and care recipients voiced the belief that integration was suitable to improve access to and outcomes for hepatitis B as well as improve convenience for both HBV mono-infected and HBV/HIV co-infected individuals.

*“It will help us hepatitis clients to get our treatment at leisure since the clinic will run from Monday to Friday and also HIV co-infected clients will save time and transport which would have otherwise been wasted for moving on two different days of the week”* Respondent 02, Hepatitis clients FGD, Koboko

Participants were unanimous in the view that the integrated care service will offer more holistic care, including counseling services which were HIV clients had experience receiving, but which was non-existent for HBV clients.

***“****The clients will get more knowledge since they will get health education on the two diseases and both clients will benefit from the education given to them.”* Respondent 11, HIV/HBV Clients FGD, Arua

### Integration to necessitate adjustments to the existing HIV care service

Many KII participants felt that integration would require modifications to some components of the existing HIV service, for a more competent system. These included patient flow processes and data systems, as noted by this Physician from Arua Hospital.

*“It is suitable if few adjustments are made because these two are high volume clinics. Patient flow through the clinics and triaging [will require adjusting] so that the triage staff can know that they are handling two groups of clients. Also patients’ data has to be integrated."* (Doctor, Arua Hospital).

Other views from KIIs regarding required adjustments included integration of client clinic visit days, training and mentorship of care providers to enhance their knowledge and skills in offering integrated care, and a phased process of integration. Participants from both Arua and Koboko also expressed that merging care of both diseases was appropriate because both diseases require similar treatment with antivirals, and also that both infections have similar transmission routes and prevention approaches. Other participants felt that community should also be sensitized regarding integration.

### Integration- a route to sustainable service delivery for HBV clients

*“I am very optimistic that this integration will bring services closer to the patients so it will be sustainable.*” Respondent 05, HIV Clients FGD, Arua

Both KII participants and FGDs of HIV clients expressed hope that integrated services will strengthen care and on-going peer support, particularly for hepatitis B clients. They revealed that HBV-infected clients would benefit from health education and counseling on a range of issues including stigma and expected duration of treatment, information that HIV-infected clients are already familiar with, to foster long term adherence.

**“***It will [most especially] favour people with hepatitis through the advice and encouragement hepatitis clients will get from HIV clients during their interactions in the facility. [It will help] them to develop more hope, live longer and also contribute to good and lasting relationship between the two parties in the clinic.”* Respondent 05, HIV Clients FGD, Arua

HIV clients felt that having been longer in the care system, they are already comfortable with it, adhering well to treatment. They however considered HBV clients as “new” to the system and they expressed willingness to offer them mentorship and on-going support along their treatment journey.

## Perceived acceptability of the integrated HIV/HBV care model

All HCWs and almost all care recipients pointed to the integrated HBV/HIV care model as suitable and acceptable to them. Reasons cited for accepting the integrated care included the free anti-retroviral (ARVs) drugs for both HIV and HBV, which all clients need for their wellbeing. Also, a possibility of future collaboration and peer support among HIV and HBV clients, as stated by this HIV-infected client from Koboko:*“I will recommend the HBV patients to come for the service because in case a HBV patient fails to come I can take for him/her drugs and they can also do the same for me another time.”* A predominantly held view was that integration will entail uniformity of systems and all services, including laboratory request forms, drug refill packages, and the ARVs themselves. This, according to them, would mask clients regarding particular disease status, making services more user-friendly and attractive. Almost all participants were willing to recommend use of the integrated care services.

### Perceived community satisfaction with integrated HIV/HBV care model

***“****It will positively impact the community because when hepatitis was at first discovered, patients were suffering and they did not know where to go. Accordingly, bringing them together will help people to get treatment hence satisfaction on the side of the patients and community at large.”* Respondent 03, HIV Client FGD, Arua

Others perceived acceptability of integrated care as dependent on overall community knowledge on the infections and the rationale for integration, as this participant from HIV-Clients FGD said- “*It will depend on one’s knowledge about these two diseases; but if majority of the population gets to know that these are diseases that [have much in] common like their medicine which is ARVs it will be accepted by the community.”* Both patients and HCWs unanimously held the view that raising community awareness about additional services rendered to HBV clients including HBV-testing, HBV-education and counseling will increase community optimism and acceptability of services. One HCW expressed the opinion that integrating hepatitis B would succeed, based on previous experience with integrating tuberculosis care into HIV care.

## Mixed perspectives about stigma

Participants expressed differing views on the influence that integrated care would have on stigma. Concerns emerged from both patients and HCWs about several issues that integration might unearth, especially stigma.

*“Of course things to do with sickness are not easy; there are people who will be surprised to see me there and it’s not good to put us together because we still have the fear of people saying we might be having HIV..”* Respondent 06, Hepatitis Clients FGD, Koboko

Though they believe that the integration approach would be the best since the two diseases share a lot in common, many participants weighed in on which of the two infections is likely to carry more stigma. Some participants believed that HBV is more stigmatizing, while many HBV-infected clients feared to be labelled as HIV-infected.

*“People who have hepatitis will say why are they bringing them to HIV clinic and yet their sickness is less stigmatising and dangerous when compared to HIV…once people see them in the HIV clinic they will say that they also have HIV, and that [is a] negative attitude.”* Respondent 05, HIV Clients FGD, Arua

### Stigma arising from the Community

Participants from most FGDs commonly held the view that once they are seen receiving care after integration, the community will judge them as dually-infected, even if they only have one infection.

*“According to me integrating us together will bring a lot of fear because some people you may meet in the HIV clinic will go and spread your name in the community that so and so who was having hepatitis is also now taking drugs for HIV in the HIV clinic there, so the issue of stigma is not going to stop.”* Participant 07, Hepatitis Clients FGD, Arua

They perceived that integration will need to be effectively communicated to affected communities in order to reduce stigma. A common view among participants was that unlike HIV, the community had low awareness about hepatitis B. They believed that the community needs information about hepatitis B, and the reason and benefits of integration, so that stigma would reduce.

### Stigma inherent within patient groups

We observed other sources of stigma inherent within the patients themselves, who held stigmatising views about their peers. Some HIV-infected patients, for instance believed that when they begin to receive the same services with HBV-infected clients, they would blame them, should their drugs run out, or should they have delays in receiving care at the clinic.

Another stigmatising view held by HIV-infected clients was that they will contract HBV when they come into close contact with HBV-infected clients within the clinic setting.

“*It will not be user-friendly especially for HIV clients because they will think they are going to get HBV since they are sitting together in the same clinic with the HBV clients*.” Participant 04, Koboko FGD

Several clients, both HIV- and HBV- infected felt that integrated care would bring all clients together and this would reduce stigma. Stigma was also viewed as an issue fuelled by limited overall community information about commonalities between HIV and HBV and the importance of integration, as stated by this HIV/HBV infected client from Arua: *“The views of people in the community cannot be predicted because so many people out there understand things differently, so views of people may vary from one person to another… but I think if people are sensitized properly about these services they will have positive [attitude] on the [integrated] services.”* Low awareness and knowledge about HBV was commonly encountered among all client groups, as well as fears among HBV clients arising from being seen in a clinic labelled as “HIV Clinic”.

Infection-status exposure was another concern for both HBV- and HIV-infected clients. Many felt that bringing them for care under one system will automatically expose their disease-specific status to peers who, without integration might never have known their status of either HIV or HBV.

## Discussion

In Uganda’s region with a high burden of both HIV and HBV, the study found that integrating hepatitis B care into existing HIV service delivery system is feasible and acceptable by both health care providers and recipients. All participants espoused the idea of offering integrated HBV/HIV care, were willing to use and to recommend use of the integrated services, contingent on requisite adjustments to the system and to the broader recipient community. Low perceived community awareness of hepatitis B and of the rationale for integration, perceived rise in stigma, exposure of individuals’ disease-status and reduced quality of services were key concerns raised by the care-recipients.

This integration of disease care systems has been consistently viewed as a beneficial strategy that boosts access to, and coverage of care for two or more chronic conditions [20–22] through coordinated inputs. In this study, both patients and providers perceived that the skills and experience gained from offering services for one condition, especially HIV, can be harnessed to upgrade care and eventually outcomes, for another. Using a coordinated approach has been echoed by Woodring and colleagues [23] in the framework for elimination of HIV, syphilis and viral hepatitis mother-to-child transmission. Similarly, the notion from participants that experience with HIV care processes would benefit and ease linkage of HBV clients to care has been alluded to by Bourgi et. al [24] in linking hepatitis C clients to care. Our findings accordingly strengthen the feasibility and end-user acceptability argument, particularly within rural SSA where HIV care systems already have significant reach.

In keeping with the theoretical framework of intervention acceptability [7], expressions from both HCWs and patients appeared to evaluate feasibility through critical reflection of elements of suitability, fitness, need and ease of integration within the prevailing context. What seemed to differ between HCWs and patients was the locus of emphasis in their dialogue. While Most HCWs judged feasibility of integrating HBV with HIV care mainly through the lens of available space, support logistics and human resource numbers and capacity, the discourse among patient groups centered on their individual needs and satisfaction with quality of services post-integration. HIV Client FGDs were especially concerned about a potential decline in the quality of services they have been receiving, and feared implications like clinic delays due to patient overload, inadvertent infection-status exposure and even drug stock-outs. A study in Kenya [25] has comparably linked higher satisfaction with service quality to increased uptake of integrated services. These concerns also corroborate those from the recipient community on scale-up of HIV care differentiation in Uganda [26], suggesting that feasibility and sustainability of integrated care will depend on quality of the integrated services. We did not observe any gender-driven differences in acceptability of care integration. This could possibly be because we interviewed individuals who had already been active in care, more keen on ensuring that there would be continuity of providing care, and therefore were more likely than not, to be accepting of changes in care processes.

Community-related issues including stigma, low awareness about Hepatitis B and misperceptions regarding the rationale for merging hepatitis B with HIV care were a basis for judging satisfaction with integrated HIV/HBV services in patient-FGDs. A minor theme that emerged in this study was the concern for infection-status disclosure in both HIV- and HBV mono-infected focus groups. Participants felt that this merged care would eventually erode their privacy regarding their individual infection-status. These issues have been recognized as having implications for continuity of integrated HIV/HBV care services [27–29]. They highlight the relevance of planning and evaluating integrated HBV/HIV care based on the local context, and inclusive of the needs and preferences of both providers and recipients [30, 31]. That care for related chronic health conditions is best merged has been theoretically supported [32]. What is less available are practical examples of how this is best achieved and sustained, in context of locally available resources and systems, a gap that our study has endeavored to close.

We note some limitations of this study. First, this study did not include all the components of feasibility and acceptability. Second, our scope was limited to care providers who serve in the clinics as well as patients in the HIV and Hepatitis Clinics. Patient care takers and facility administrators were not interviewed. This therefore did not capture broader social support, institutional governance or policy-related views on HIV/HBV care integration. The study nonetheless had notable strengths. We used mixed focus groups and key informants to capture views of providers and care recipients. This accorded us the opportunity to capture diverse views, opinions and reflections on perceived benefits, costs, needs and preferences in judging appropriateness and acceptability of HIV/HBV integrated care processes. To further improve data validity, we used both key-informant interviews and focus groups to obtain views from imminent providers and recipients of HIV/HBV integrated care.

In conclusion, our study has showcased potentially beneficial and acceptable integrated response to two infections with commonalities across disease natural history, transmission, care, treatment and prevention. Patients would receive a more efficient service, as HCWs become competent in managing both conditions at primary care level. In addition, HBV infected individuals would receive a more holistic care comparable to that received by HIV-infected persons, if communities are educated about HBV prevention and the rationale for providing integrated care.

## Supplementary Information

**Additional file 1.** Feasibilityand Acceptability of HIV and HBV care integration FGDCoding matrix on Patient andHealth care worker perceptions.

**Additional file 2.** COREQ (COnsolidated criteria for REporting Qualitative research) Checklist.

## Data Availability

The data supporting the conclusions of this report have been included within this article and in additional files 1 and 2.
