# Supplementary material for: Feasibility and acceptability of integrating hepatitis B care into routine HIV services: a qualitative study among health care providers and patients in West Nile region, Uganda
Source: BMC Health Serv Res. 2023 Jan 20;23:59. doi: 10.1186/s12913-022-08924-0 (PMC9854069; doi:10.1186/s12913-022-08924-0)
Supplement: Supplementary file 1 — Additional file 1. Feasibilityand Acceptability of HIV and HBV care integration FGDCoding matrix on Patient andHealth care worker perceptions. [file 12913_2022_8924_MOESM1_ESM.docx]

**Feasibility and Acceptability of HIV and HBV care integration**

**FGD Coding matrix on Patient and Health care worker perceptions.**

| **Theme** | **Sub theme** | **Category** | | |
| --- | --- | --- | --- | --- |
|  |  | **HIV Clients** | **Hepatitis clients** | **Integrated HIV/Hepatitis clients** |
|  |  | **Issues arising/ codes** | **Issues arising/ codes** | **Issues arising/ codes** |
| SUITABILITY OF THE HIV/HBV INTERGRATED CARE SYSTEM | Meaning of Integrated HIV/HBV care | - Integration means trying to see how they can bring people of HIV/HBV together for their treatment in the same place. (FGD HIV clients Koboko) - Integration means trying to see how they can bring people of HIV/HBV together for their treatment in the same place. | - To me I think it is bringing the two people together because they are all taking the same drugs. Therefore, it will need to be spread through sensitization in order to ensure that it works. (FGD Hepatitis clients Koboko) | - It means combining HIV and Hepatitis together for better management. *(FGD HIV/HBV clients_Koboko)* |
|  | What it encompasses | - The number of staff should be increased first before starting the integration since number of the clients is going to be very high and more people are going to come for the services. There is also need for proper and consistent health education to ensure that the two parties are aware that they are going to be together especially in the facility and also over the radio. (FGD HIV clients Koboko) - I think also it will involve counselling hepatitis clients first before integrating us together with them since counselling is the first way to good drug adherence. (FGD HIV clients Koboko) - It will be good to bring us together because the hepatitis clients will also get counselling services here together with us in order to hope for themselves since hepatitis is very much stigmatizing in nature. |  |  |
|  | Suitability of integrated HIV/HBV care system | - This integration will be suitable because it will bring services nearer and closer to the patients and so the Government should work in collaboration with the religious leaders in order to help sensitize the people in the communities about the integrated service since people in the villages trust more the religious leaders. (FGD HIV clients Koboko) - **Respondent 7.** In terms of the issue of human resource, it will save the hospital since the few staff will help in serving the big population under one roof. (FGD HIV clients Koboko) - This integration will be suitable because it will bring services nearer and closer to the patients and so the Government should work in collaboration with the religious leaders in order to help sensitize the people in the communities about the integrated service since people in the villages trust more the religious leaders. - The ways of getting the diseases is all the same and their drugs are also the same ,so it will be easier to get their drugs since they are all kept in the same pharmacy. - In terms of the issue of human resource, it will save the hospital since the few staff will help in serving the big population under one roof. | - It will only be advantageous for the hepatitis clients because they used to have one day a week for their treatment and of which once you missed you wait until the next week so integrating will favour them because they will get treatment in any of the days of the week. (FGD Hepatitis clients Koboko) | - If the two clinics are integrated it will be suitable for hepatitis clients because they will receive treatment throughout the course of the week instead of only attending clinic once a week. *(FGD HIV HBV clients_Koboko)* - The services can be offered in the same facility only that some rooms should be allocated inside there for hepatitis and HIV clients in order to handle them differently because these two clients also speak in different languages however much they do get the same medicines. *(FGD HIV HBV clients_Koboko)* - It will be suitable because of human resource challenges in the hospital since staffing is not enough and secondly for one to accept to be integrated it means the client to a greater extent has left the worries of stigma only that more health education is needed for these two clients in the facility in order to make them fully understand the importance of the integration gradually. *(FGD HIV HBV clients_Koboko)* - It will be suitable if the clinic day for hepatitis is run from Monday to Friday in order to reduce the overwhelming population in the clinic since the space in the clinic is not going to be enough. *(FGD HIV HBV clients_Koboko)* |
|  | Positive effects of the Integrated HIV/HBV care system | - It will favour people with hepatitis because through the advice, and encouragement hepatitis clients will get from HIV clients during their interactions in the facility hence making them to develop more hope, live longer and also contributing to good relationship between the two parties in the clinic. (FGD HIV clients Koboko) - It will favor people with hepatitis because through the advice and encouragement hepatitis clients will get from HIV clients during their interactions in the facility hence making them to develop more hope, live longer and also contributing to good relationship between the two parties in the clinic. | - It will help hepatitis clients to get their treatment at leisure since the clinic will run from Monday to Friday and also for mono-infected clients they will save time and transport which would have otherwise being wasted for moving in two different days of the week. (FGD Hepatitis clients Koboko) | - It will create good relationship between hepatitis clients and HIV clients since they will know that they are all taking the same medicine. *(FGD HIV HBV clients_Koboko)* - It will make work easier for the few health workers and also stigma will be reduced among the clients. *(FGD HIV HBV clients_Koboko)* - The clients will get more knowledge since they will get health education on the two diseases and both clients will benefit from the education given to them. *(FGD HIV HBV clients_Koboko)* - One stop shop kind of treatment will reduce time wastage of moving to get treatment from different places and also it will save human resource. *(FGD HIV HBV clients_Koboko)* |
|  | Negative effects of the Integrated HIV/HBV care system | - People who have hepatitis will say why are they bringing them to HIV clinic and yet their sickness is less stigmatising and dangerous as compared to HIV so once other people will see them in the HIV clinic, they will say that they also have HIV so that negative attitude will there in people for them. (FGD HIV clients Koboko) - Hepatitis clients will complain of wasting of their time in the HIV clinic since they are very few and can be seen quickly by the doctor and yet they will also be meant to wait for long hours like for those with HIV. (FGD HIV clients Koboko) - In case of the shortage of drugs the HIV clients will think hepatitis clients are the ones finishing their drugs. (FGD HIV clients Koboko) - Work overload will be too much on the few health care workers because of the increased population taking treatment from the facility if more staff is not added. (FGD HIV clients Koboko) | - According to me integrating us together will bring a lot of fear because some people you may meet in the HIV clinic will go and spread your name in the community that so and so who was having hepatitis is also now taking drugs for HIV in the HIV clinic there, so the issue of stigma is not going to stop. (FGD Hepatitis clients Koboko) | - The issue of stigma will still prevail because what an HIV positive and Hepatitis clients will think of after seeing a colleague taking treatment from this clinic will not be of a good perception and also if equipment for investigations like the ultra sound machine are not present it will not be suitable. *(FGD HIV HBV clients_Koboko)* - It will need that the number of clinicians and other health care workers to be increased because the population of the integrated clinic will over whelm the number of the staff on ground. *(FGD HIV HBV clients_Koboko)* |
|  | Appropriateness of the “integrated HIV/HBV care” system | - According to my observation, this integration is going to affect the hepatitis clients most because for us HIV clients we have become used to our system due to the health education being given to us since hepatitis clients have not being sensitized concerning the issues relating to their sickness ranging from stigma up to the period for treatment, so they will have more stigma. (FGD HIV clients Koboko) - It will be appropriate because of the nature they are contracted and treated for the Government it will reduce costs and also help to capture all the clients together easily especially for testing both diseases. (FGD HIV clients Koboko) - I think the name of the clinic that is written up in front of the facility that is HIV CLINIC alone is first of all going to suppress the minds of the hepatitis clients because they will say why now bringing them in the HIV clinic and yet they are not having HIV so this name alone is going to increase the level of stigma among the hepatitis clients if it is not changed before the integration. So will suggest that the name should be changed as INTEGRATED CLINIC to reduce stigma. (FGD HIV clients Koboko) | - The best option to me will only be changing the name of the clinic from HIV to ART CLINIC in order to make it to be appropriate in terms of bringing us together. Secondly, clients should come freely on any day of the week to pick up their drugs in order to decongest the increasing population in the facility. (FGD Hepatitis clients Koboko) - It will only be appropriate if more health care workers are empowered through some training in order to increase the number of the existing staff serving the clients this is because since i started medication i have never been counselled, so integration will help the hepatitis clients to get more hope through the counselling services in the clinic because most times we have just being counselling ourselves as patients alone. (FGD Hepatitis clients Koboko) | - When enough information through health education and counselling is given to the patients and also the reason for integrating these two clinics is properly explained to the clients it will be comfortable for them to use. *(FGD HIV HBV clients_Koboko)* - The way health workers are going to conduct themselves in the clinic will also influence the client’s mood in receiving the integrated service basically health workers should show more love and care for the patients. *(FGD HIV HBV clients_Koboko)* |
| SATISFACTION | Satisfaction with the integrated HIV/HBV care system | - I will only be satisfied if everything that is needed for the integration to move on well are all in place especially training of the staff to get knowledge on the management of hepatitis because not all HIV staff here are all aware about hepatitis, enough sensitization and space for files and also more chairs for sitting, if these are in place we shall be satisfied. (FGD HIV clients Koboko) | - I will only be satisfied when I am counselled and get quick services in the integrated care service plus the health education which we hepatitis clients used not to get. (FGD Hepatitis clients Koboko) | - I will only be satisfied if there is proper health education and counselling being given to the clients. I will also be satisfied with the integration because HIV has the greatest stigma, so they will also gain some hope if they are brought together. *(FGD HIV HBV clients_Koboko)* - I will be satisfied if all the health workers are empowered through facility-based training about the two diseases such that they can deliver better services to us the patients. *(FGD HIV HBV clients_Koboko)* |
|  | Dissatisfaction with the integrated HIV/HBV care system | - If the number of health care workers is not increased and the time for patients is wasted because of long hours spend while lining for treatment, hence I will not be satisfied. (FGD HIV clients Koboko) | - If people are so many and there are few health care workers giving treatment and you remain being un attended to with the time wasted I will not be satisfied with the integrated care system. (FGD Hepatitis clients Koboko) | - First come, first served should be the order of the day, so if hepatitis clients are served first because they are few in number I will not be satisfied with the services. *(FGD HIV HBV clients_Koboko)* - I will not be satisfied if the name of the clinic remains as HIV clinic. *(FGD HIV HBV clients_Koboko)* |
|  | Community beliefs about the service |  | - It will not be user-friendly to mix HIV and Hepatitis clients together because of the HIV stigma. (FGD Hepatitis clients Koboko) - It will depend on ones knowledge about these two diseases, but if the majority of the population gets to know that these are diseases that do have many common things together like their medicine which is all ARVs it will be accepted by the community. (FGD Hepatitis clients Koboko) | - I think some people will not believe in this integration because some people in the community will not be happy and say how can ARVs be given for the hepatitis clients and yet they are not HIV positive. *(FGD HIV HBV clients_Koboko)* - Some people will believe in the services if only sensitisation is done adequately hence it will make the community to understand the need for integrated service. *(FGD HIV HBV clients_Koboko)* |
|  | Views of people in community about satisfaction with HIV/HBV integrated care system | - It will have positive impact in the community because when hepatitis was at first discovered, patients were suffering and they did not know where to go, so bringing them together will help the people to get treatment hence satisfaction on the side of the patients and community at large. (FGD HIV clients Koboko) | - As long as you explain to the community through sensitization that though they are different sicknesses but both take the same medicine that is ARVs the community will be satisfied with the integrated care service. (FGD Hepatitis clients Koboko) - For me I see once the name of the clinic is changed people will accept it easily coupled with continuous sensitization the minds of the people will open to embrace the integrated care service. (FGD Hepatitis clients Koboko) | - The views of the people in the community I cannot predict it because so many people are there outside and they understand things differently so the views of the people may vary from one person to another so not all will agree or disagree with the integration but I think if people are sensitized properly about these services, they will have positive take on the services. *(FGD HIV HBV clients_Koboko)* |
| INTENTION TO USE SERVICE | Willingness to use Integrated care service | - I do accept the integrated service because birds of the same feather move together since we are all taking ARVs. (FGD HIV clients Koboko) | - I am very ready to start using this service as soon as the name of the clinic is changed to the INTEGRATED CARE CLINIC. (FGD Hepatitis clients Koboko) | - To me it should not start immediately because it is yet abrupt so people need to be sensitized properly first such that they can understand how this program will benefit them. *(FGD HIV HBV clients_Koboko)* - I believe it should take some little time like two months for sensitizing both the HIV and Hepatitis clients first with some of the things we said needed tobe put right in place first before we go integration. *(FGD HIV HBV clients_Koboko)* - For me I agree that it should be started immediately because when a new development is to be brought whether you sensitize people or not there will still be a lot of resistance because people in most cases fear change. *(FGD HIV HBV clients_Koboko)* |
|  | Intention to use the integrated care service | - We are willing to use this integrated service. (FGD HIV clients Koboko) | - Yes, we intent to use it. (FGD Hepatitis clients Koboko) | - Yes when everything is put in place, it can be started. *(FGD HIV HBV clients_Koboko)* |
|  | Whether Integrated HIV/HBV care service will be used by patients / clients seeking Hepatitis B care services | - They will accept because they know we all have common problems and the only solution is here in the integrated facility. (FGD HIV clients Koboko) - They will accept because the treatment is free being given by the Government so if you are to buy the medicine out there it is very expensive. (FGD HIV clients Koboko) | - If they get enough sensitization, they will use it. (FGD Hepatitis clients Koboko) | - They will come once it is started immediately because they need treatment. Some people are going to be worried when they are identified taking drugs together with the HIV clients which will cause a lot of problems among the people in the community. *(FGD HIV HBV clients_Koboko)* - If the name of the clinic is changed from HIV CLINIC to may be INTEGRATED CLINIC more people with hepatitis will come for services because they are sick. *(FGD HIV HBV clients_Koboko)* |
|  | Recommendation for people in the community to use the Integrated HIV/HBV care service | - Yes, I will recommend the integrated care system for all the people out there. (FGD HIV clients Koboko) | - Yes we shall recommend the integrated care services to be used once everything we need is in place. (FGD Hepatitis clients Koboko) | - Yes, we shall recommend people to use the integrated services as soon as we see its importance but as of now it has not yet started so we want to see it working first before we do recommend it. *(FGD HIV HBV clients_Koboko)* |
|  | Readiness to change to Integrated care service | - If possible, I wanted this program to start immediately since we have people already infected and they need treatment. (FGD HIV clients Koboko) - Hepatitis clients need to be sensitized for at least two months first before they are integrated into the HIV care system in order to reduce stigma. (FGD HIV clients Koboko) | - We are very ready to change even if it is to start now. (FGD Hepatitis clients Koboko) | - If all the things we have said we want them to be in place are put right yes we are going to be ready for the integrated care service. *(FGD HIV HBV clients_Koboko)* |
|  | Likelihood that the integrated service will be sustained? | - If the number of health care workers is not increased and yet people are sensitized about the services and the population coming for treatment is bigger than the staff number it will not be sustainable. (FGD HIV clients Koboko) | - This integrated service will only be sustainable if the number of the health care givers is approximately equal to the population that is going to receive the services and everything needed for the integration is in place like appropriate training for the staff and drugs are all in place including the ultra-scan system which is used for some check-ups so definitely it will be sustainable because human resource is enough including drugs. (FGD Hepatitis clients Koboko) | - If the services are offered maximumly and consistently with everything being put right in place this program will be sustainable. *(FGD HIV HBV clients_Koboko)* - If all the implementing partners like the Government, IDI, and MSF come together and make a good memorandum of understanding to see that they all embrace the integrated care service it will be sustainable because they will all be working to see one common goal is achieved. *(FGD HIV HBV clients_Koboko)* |
|  | Other emerging views | - What if TB is also being brought here because hepatitis and HIV are integrated will it be bad because some HIV clients are also taking TB drugs? - We HIV clients are aware that we taking our medicine for life, so for hepatitis want to know whether they can be cured with their treatment or they also take drugs for life like for HIV? (FGD HIV clients Koboko) | - In some places clients like HIV/Hepatitis clients are being supported in groups in terms of finance and some income generating activities in order to support themselves in their treatment by some implementing partners and even the Government, so I wanted to know whether such a package will also be organised for us. (FGD Hepatitis clients Koboko) - My view is that if possible those health care staff who are Hepatitis positive should be brought here in the facility to help in counselling us since they have more experience of the sickness than us such that we can also develop some kind of hope for us since hepatitis is now being perceived as the most killing disease as of now worldwide. (FGD Hepatitis clients Koboko) | - For me I want to know whether they are going to make some files for the hepatitis clients and if so, their files should be put separate from those of the HIV clients to avoid mixing them up in the triage. *(FGD HIV HBV clients_Koboko)* |

**KII Coding matrix on the Feasibility of HIV and HBV care integration: Patient and Health care worker perceptions.**

| **Theme** | **Sub theme** | **Issues arising/ codes** |
| --- | --- | --- |
| **SUITABILITY OF THE HIV/HBV INTERGRATED CARE SYSTEM** | Meaning of Integrated HIV/HBV care | - Having both clinics run under the same program using the same processes since they have many things in common like transmission, management and treatment using the same drugs. *(Dr. Wilson HIV clinic ARRH)* - Bringing all these services together under one roof and using the same staff for different patients. In the maternity, it will focus on the status of the mothers so as to enable the health worker handle expectant mothers with care in order to prevent transmission from mother to child. We test all the mothers for HIV, for hepatitis it is done in the laboratory and they just bring for us the results and again for hepatitis we have not been so much key on the on its test for the mothers however, for HIV we have been so keen on it and at least for every mother we are bothered to do so to reduce MTCT of the diseases. *(ADIRU NURSE ARRH)* - This is where services are offered as a package in one setting or by one person unlike offering services at different sites by different people, as one person presents the service can be offered by one person. *(ADMINISTRATOR ARRH)* - It is like a one stop centre for Hepatitis and HIV services, running Hepatitis and HIV together in one setting I think it is something long overdue and something we should have had long ago. Right now in our facility we have integrated hepatitis clinic into the HIV clinic and this means all the hepatitis clients are being reviewed from the HIV clinic and those who need treatment will be treated from there but the entry point is the OPD, all the newly diagnosed, whether referrals from outside or diagnosed within the facility will have to pass through OPD basically because of the data tools that are not there, therefore, we always endeavour to capture all the hepatitis clients in the primary tools. *(Clinician koboko hospital)* - To me I see it more effective in that we are going to use the same staff in HIV service to provide the same hepatitis services. Uniquely these are viral diseases and it’s true that most likely people who have knowledge on hepatitis have also on HIV and hepatitis and HIV have similar mode of transmission and the staff all have the basic knowledge and therefore, the service can easily be offered by these staff from HIV and hepatitis clinics. *(Clinician Koboko hospital)* - Secondly, the logistics like the drugs we use tenofovir which is a component of drugs used for treating HIV and the staff in the HIV clinic know the side effects, how tenofovir can be taken, who do not qualify for tenofovir and those who do qualify for it and i think it is something so nice, therefore, it is very effective to have these two clinics being run together in one setting and also the issues of few staff, limited space and the technical knowledge hence it be good to integrate. *(Clinician Koboko hospital)* - Integration means a lot to me, as we said Hepatitis and HIV are related in so many ways, that is in terms of their transmission, and their treatment, and monitoring are all the same and for the two liver function tests are all to be done with viral load. *(INCHARGE HIV CLINIC ARRH)* - Putting together or inserting already into an existing process that is HIV and Hepatitis being carried together co-currently at the same place and time. Although Hepatitis and HIV are related, but it takes some expertise that is to manage these two diseases because I think not all those who are managing HIV have perfect knowledge in managing Hepatitis that is in terms of management and may be mode of transmission unless someone is trained and given the necessary empowerment. *(Sr Susan ARRH)* - This is where two entities that were functioning separately and now having them to function together if they were under different roofs now, we want to have them under one roof. For example, HIV and Hepatitis all had different structures now the two are going to be under one roof, the same staff coming together in the same place and are going to take care of the different patients together under one roof. *(MARY Laboratory ARRH)* - This is where services that have been given parallel come to be delivered in one setting and in the same facility by the same people for the purpose of better service delivery. It can be at the level of the clinic itself where consultations and dispensing can be done and they all use the same laboratory. *(HOSPITAL DIRECTOR ARRH)* - It is a one stop model of offering hepatitis and HIV services together in one clinic where if you are handling a HIV client you can also manage the patient if he also has hepatitis. Integration for a holistic management will mean offering both services completely in the same place. Here managing hepatitis and HIV requires a team of health experts or health workers where at one level of management if someone is stuck can be able to consult another person. *(ISAAC clinician ARRH)* - Putting together all under one umbrella diseases which are not related but similar to HIV and managing them together under one roof. (*Teddy HIV clinic data ARRH)* |
|  | What it encompasses | - It encompasses all the systems ranging from testing, registration, counselling and its management and this integration involves all these systems to be in place*. (Dr. Wilson HIV clinic ARRH)* - It will also involve expansion of the health workers knowledge since it is given as a package, the health worker will be equipped with knowledge of the two diseases. *(ADMINISTRATOR ARRH)* - This integration will encompass offering the services in the same setting, probably in the same days of the week, by the same people who should be able to have the skills for offering the services, and the patients come mixed and sit together while they receive the services. *(HOSPITAL DIRECTOR ARRH)* - It will involve a lot, there is logistics needed, supply of medicines, items in the laboratory, viral load tests, where there is a proper channel for doing viral load like in the HIV setting where after collecting blood sample from the client it is sent to CPHL, therefore, hepatitis would also need a system like that. It will also need medical personnel here to help us in terms of consultations and I do not know whether in the side of IDI artic will also help in case we get challenges with the hepatitis clients. *(ISAAC clinician ARRH)* - I think before you talk about the system, you have to talk about the procedures like Human resource and then what will the whole flow be like and talk about the data. (*Teddy HIV clinic data ARRH)* |
|  | Suitability of integrated HIV/HBV care system | - It is suitable if few adjustments are made because these two are high volume clinics like floor of the clinics and triaging such that the triage people can know that they are handling two clients. Also, data has to be enlarged. *(Dr. Wilson HIV clinic ARRH)* - The number of the clients is not all that much since hepatitis management is not like for HIV where you need to be started on treatment immediately, but with hepatitis someone goes through further evaluations so they are not eligible for treatment until further notice. Though the number may look big, but not all of them will go for treatment since the actual number for treatment will be small *(Dr.wilson HIV clinic ARRH)* - Changing the name to integrated clinic would make the patients to feel more comfortable because whoever enters there goes for an un defined care. *(ADIRU NURSE ARRH)* - The service delivery is going to be good because in the HIV clinic there are patients who have hepatitis and are sent for investigations they bring and some are put on ARVs for hepatitis and yet these drugs are in the HIV clinic, however, if all these drugs are under one roof it will be suitable since they all take ARVs. *(ADIRU NURSE ARRH)* - It will work since the services are currently offered at different sites; however, the integration will make the services be offered in one facility which will reduce the burden of space shortage in the hospital and putting the services under one roof. *(ADMINISTRATOR ARRH)* - It is good but what i see as a road block is the pre-existing services which were already on ground in the HIV clinic .The challenges which we already had in the HIV clinic will affect the performance of the integration especially staffing challenges since here there is a lot of documentation and on top Hepatitis is another additional work where registers are so many and where there is no data ,there is no work done therefore, what I wanted is once it is integrated there should be a clear data system. This integration is going to be suitable is because these are related diseases especially in terms of their treatment, doing viral load, monitoring and patient follow up. *(INCHARGE HIV CLINIC ARRH)* - In principle it is actually suitable, why I say so is when you look at one side especially the HIV clinic ,it is well organised and it is a matter of going to absorb hepatitis to it without leaving any big input in terms of resources because counsellors we already have enough and health educators, although the challenges we not reach gap and we only have to give them more information on the disease which they have not being taking care of well. *(DR. OMALE ARRH)* - In my opinion I take it positive and suitable in terms of scarce resources for example, here in the hospital, we run so many specialised clinics in different days and you realise that the same personnel are the ones running these clinics and sometimes they get over whelmed at the beginning and the next days you will see a different scenario. *(Sr Susan ARRH)* - I feel it would be a good thing to do because the two are viral diseases, the Doctors working on both hepatitis and HIV have the knowledge on the two viruses, so the Doctors are well versed with these two diseases and i think managing them under one roof will really work very well and also it will make work easier for the health workers. Stigma wise, I believe if the hepatitis clients join those in the HIV clinic the stigma will go because it is just a new thing as compared to HIV whose stigma has been for long and up to date. *(MARY Laboratory ARRH)* - It can be suitable for me from the care givers perspective since they are not given different treatment days, it would have reduced the number of separate clinic days which have to be seen by the few clinicians and doctors in the hospital. For the recipients of the service, and for the patients who are co-infected it will save them since the different diseases of the patient can be confronted by the same physician at the same day. *(HOSPITAL DIRECTOR ARRH)* - These two activities can work in the same place when we address certain things like having a container for files, then some space or days to be designated for hepatitis during the five days of the week because here the clinic runs daily from Monday to Friday, maybe we can choose 2 or 3 days in a week so that they can run together. It would be suitable to combine if all what we desire is there. *(ISAAC clinician ARRH)* - The Integration in our view is well come but still you have to get the opinion of the clients themselves being under one roof you have to get the broader spectrum from the personnel here because at the moment we are just too over whelmed here and that might need more staffing and I do not know whether your planned to merge the Staff from Hepatitis clinic to those here in the HIV Clinic. For the case of the clients, I cannot give a stand on that because I do not talk to the clients since I do not know the language and really do not know what the clients will feel like. For the case of the Healthcare workers, I think it will be okey because once there is intermarriage of information like people of HIV are taught how to manage Hepatitis and Vice versa but still the work load is big. As a data person I work with system, there is need for a system to manage it like merging the two together therefore in the system we shall just have to log – in one screen and do that as one stand instead of having a parallel system. Therefore, it possible and I am ready for it as long as you give me more staff. (*Teddy HIV clinic data ARRH)* |
|  | Positive effects of the Integrated HIV/HBV care system | - There will be generally positive effects this integration will bring, since the HIV setting has a good system like counselors and testers and this will improve hepatitis management like adherence and issues of lost to follow-up will be manageable. *(Dr. Wilson HIV clinic ARRH)* - It will help to increase the rate of service delivery since the patients will be served timely because they are all seen by the same clinicians instead of being seen on different clinic days. *(ADIRU NURSE ARRH)* - It will also help the patients in a way that, they will be seen throughout the week not only on the specified clinic days because there are some who are diagnosed on Friday and you tell them to come on Wednesday during the next clinic day, but if it is integrated will serve the patients all at once. *(ADIRU NURSE ARRH)* - The knowledge and skills of the service provider should be able to be enhanced by the training or mentorship that will come with the integration. Am looking at a situation where the integration will not just drop like a bomb without telling people what to do and what they are going to do. *(ADMINISTRATOR ARRH)* - The package of knowledge and skills sharing should be able to enhance the health worker to perform even better, the stress element I talked about of work space should not be talked about because these two diseases are going to be managed under one roof. *(ADMINISTRATOR ARRH)* - There will be proper utilisation of time, it will shorten time for service delivery because a clinician sees you as a package instead of seeking treatment from different clinics. *(ADMINISTRATOR ARRH)* - We would have minimised on the issue of space and how the two would have been run differently means we are going to occupy the two spaces however, when they are run together under one roof, the space for hepatitis will be used for another program. *(Clinician Koboko hospital)* - These diseases are related especially in terms of viral load tests and teir treatments all use ARVs hence managing them in the same facility will be very easy. *(INCHARGE HIV CLINIC ARRH)* - Secondly, lost to follow-up of these clients of HIV and Hepatitis, so if it means follow up of these clients especially oral visits in their respective places of residence it will be easy since you may have patients to follow up and are all in the same route regardless of their conditions so managing such patients will become very easy instead of following clients in different locations who may be difficult to access in the same time. *(INCHARGE HIV CLINIC ARRH)* - There are some clients who also need counselling because of some social and psycho-social issues and some have already lost hope and need to be counselled especially some hepatitis clients. Therefore, the integration will mean Hepatitis clients will also be managed like HIV clients hence satisfaction especially by hepatitis clients because of quality service. *(INCHARGE HIV CLINIC ARRH)* - Integration reduces the burden of single disease management since experts are able to handle two or more infectious diseases at the same time under one roof, so, a team of very few can ably manage more diseases compared to handling diseases single handily given the human resource factor. *(DR. OMALE ARRH)* - Integration will help us to know the adherence of our clients hence right action will be taken. *(DR. OMALE ARRH)* - Data management will improve since the HIV clinic already has a software system for capturing data and therefore data analysis will be very easy. *(DR. OMALE ARRH)* - The positive effect is that both diseases present themselves with stigma so initially, when we started managing HIV condition, we had a specified point where someone would go and not until we started integrating HIV in other services for example in OPD, Dental, and MCH a person is tested for HIV and we had counsellors in these areas to handle them and to me the idea of integration is very perfect. *(Sr Susan ARRH)* - With the HIV clinic they have those timely programs and it has already being, there, hence with the integration, people will receive smooth and quality care timely. *(MARY Laboratory ARRH)* - The integration will come with a lot of benefits such as stocking of reagents especially for the laboratory and other equipment also new staff will be added onto the existing one hence addressing the challenge of few staff. *(MARY Laboratory ARRH)* - The positive outcome especially for the patients is that they can spare time especially the extra time for having different clinic days. *(HOSPITAL DIRECTOR ARRH)* - There will also be service efficiency since the different diseases are worked on together at the same place like one stop shopping centre like in supermarkets and at the same time it will help to address issues of stigma. *(HOSPITAL DIRECTOR ARRH)* - It will be suitable because it will reduce work load since staffing is not enough hence closing the gap in staffing and in terms of human resource it will save us. *(ISAAC clinician ARRH)* - It will reduce time for a patient to be managed instead of a patient being handled in different locations, he/she will be handled holistically just in one place. *(ISAAC clinician ARRH)* - Integration always comes with easy data management; you know there is data readily available and you can easily follow up a client. (*Teddy HIV clinic data ARRH)* |
|  | Negative effects of the Integrated HIV/HBV care system | - However, the negative effect will be that the population received for both HIV and HBV patients will be overwhelming number to the healthcare workers which I think they will complain of the work over load. *(Dr. Wilson HIV clinic ARRH)* - There is one thing that kills the community, that is stigma, it would have been a good idea to integrate the two, but then what kills the community is then the stigma aspect of it, however, if they learn that hepatitis treatment is being transferred to the HIV clinic, some patients who would want to seek care will tend to withdraw because of the stigma of being identified in the HIV clinic and that is the only thing that will be set in the mind of the people in the community, but otherwise ,it would have been a good thing to reduce on the work load so as to have a better service delivery. *(ADIRU NURSE ARRH)* - May be the flow of the patients will be overwhelming, the staff might be over worked in terms of hours than the usual clinic days. *(ADIRU NURSE ARRH)* - When introducing integration, people think that is additional work in their mind set, people will say I have been handling this, now they are bringing this once again, naturally and humanly you will think that is additional work, therefore, there should be a deliberate effort to demystify that this is just another service it is not going to increase on the work load. Therefore, to me this is another fear that will be attached with the integration. *(ADMINISTRATOR ARRH)* - Human expectations especially for some allowances or increase in pay, you know we health workers we getting so much oriented to money and probably rightly so because of what we are going through and this is also another fear i see will be there. *(ADMINISTRATOR ARRH)* - Also, the fear of the un known because we are not aware of the direction this integration will take, will it work, will the service be of quality, will it not be overwhelming to us, as such at some point will it not collapse. *(ADMINISTRATOR ARRH)* - The fear is that hepatitis is now being added to HIV clinic so there is going to be some interference in the flow of work, therefore with these fears the people should not be blamed initially. What the policy says because the study is trying to find out whether it really works because we want it to work, so as the study goes, will there be a policy developed to make sure that it is escalated across the whole country. *(ADMINISTRATOR ARRH)* - The client load will grow and will also over whelm the resources we have on ground especially the staff, infrastructure and even the logistics will not be enough to provide the care that will be needed. *(Clinician Koboko hospital)* - For the clients we shall need a lot to make them understand since some will not be basically having HIV and yet they are coming for other services. Therefore, a lot of health education is needed to create awareness among these clients about the reason for the integration and importance of this in order to make them embrace it. *(Clinician Koboko hospital)* - More sensitization through peers, because the use of peers is more effective than using staff in order to help them talk to their fellows and the community and then the community can easily view them as people who are better on treatment and lastly, the name of the clinic should be changed as infectious diseases clinic to reduces on the levels of stigma among these clients. *(Clinician Koboko hospital)* - Since the HIV clinic is a high-volume facility and we are going to use only the available human resources, work load is going to be high and integration is therefore already an extra work on the existing few staff in the facility. *(INCHARGE HIV CLINIC ARRH)* - On the workers side I believe there should not be any acceptability problem because when it is based in the hospital, they are going to work on patients, therefore, I do not expect the workers to just say this belongs to that, however, the challenge then was that the HIV clinic was managed by partners who mandates the thought was only HIV, little knowing that this was also a problem on their clients than they faced. So, the problem was that they did not want anything outside HIV. *(DR. OMALE ARRH)* - It will be more of a challenge but not really negative in that case because may it will just be the number of the clients who are going to be seen by a health care worker which will be over whelming to a health worker. *(Sr Susan ARRH)* - Secondly, why we have managed HIV is because we now have many partners helping us to manage the clinic and when HIV is going to be integrated, we shall see both diseases and if you take the space in the clinic is not enough to accommodate the ever-increasing population that is seeking for services and therefore more resources shall be required to improve the facility base. *(Sr Susan ARRH)* - For us in the laboratory I don’t think there is anything, negative but we see it is going to be in the clinic site where they will be over whelmed with a lot work and also the population is going to be too big for them, otherwise, from our side we may only experience cases of stockouts and equipment breakdowns like the chemistry machine, but when it comes to staff, our staff can handle it well. *(MARY Laboratory ARRH)* - There will be work over load for health workers since more patients are seen in a given day as compared to the different clinic days and this will also in turn increase workers resistance. *(HOSPITAL DIRECTOR ARRH)* - Stigma will still be on ground especially for hepatitis clients who will still be fearing to be identified taking treatment from the HIV clinic. *(HOSPITAL DIRECTOR ARRH)* - Stigma cuts across the two diseases, so the fact that a hepatitis client will fear being spotted in the ART clinic and yet he/she is not HIV positive, so this will pose stigma. *(ISAAC clinician ARRH)* - The number of clients going to be seen is going to increase completely, because in ART clinic the number we see averagely on daily basis is from 150 to 200 clients, except on Tuesdays and Fridays where the number may be lower than that, so the number might go high hence more work. *(ISAAC clinician ARRH)* - Clients will spend more time and we shall need to change the way of making appointments. The whole team also needs to be empowered as we are going to work as a team such that I will be very conversant with the work such that the integration can take place. *(ISAAC clinician ARRH)* - People’s perspective like the clients are they willing to embrace it to like having them under one roof, that is why I said like in the beginning how are the procedures you are going to use like going to have them all under one roof or seeing they are run parallel or in different days. (*Teddy HIV clinic data ARRH)* - Stigma is a big factor but I think if clients are sensitized every day it will be reduced that is why our clients here are seen in different days like the children, adults and adolescents because of the stigma part of it and I don’t know how you really want to come up with it. As long as the stake holders are put in the loop right from the initiation, I think it won’t fail. (*Teddy HIV clinic data ARRH)* |
|  | Appropriateness of the “integrated HIV/HBV care” system | - People's main fear is the number going to be received in the clinic, however, with hepatitis few people will be managed. Community drug refills always help to decongest the numbers in the clinic, so if for hepatitis is started it will reduce work over load. *(Dr. Wilson HIV clinic ARRH)* - It will be good because, if at all, this patient is tested for hepatitis and he/she is positive, it will be easier for the patient to adhere to treatment since he will be taking drugs for two things at the same time hence making him to take drugs consistently. *(ADIRU NURSE ARRH)* - For a hepatitis client it is going to be very easy to access the clinic, doctor, and any clinician in the course of the week if he/she is tested positive with hepatitis. *(ADIRU NURSE ARRH)* - Once the service provider is empowered with knowledge and skills to manage both it will translate into improved service delivery. So, integration will not be just by words because it will be in position to strengthen the health worker to actually provide a more comprehensive service because of the empowerment this person will have got. *(ADMINISTRATOR ARRH)* - I think the hepatitis and HIV co-infected patients will benefit more than any other client because they will be able to receive all their treatment under one roof and on time. So, when we integrate, we shall have to identify those co-infected and put them to benefit from the routine checks therefore, they will be more comfortable because there is more stigma in HIV than in hepatitis so if hepatitis is merged into HIV care they will be comfortable. *(Clinician Koboko hospital)* - It is appropriate, but always when something is initiated newly there are challenges but with time, we will overcome them. The problem that is going to arise is may be work over load on the few staff but if staff are there will be no headache and the clients will get the services as usual without complain. *(INCHARGE HIV CLINIC ARRH)* - For this integration to be appropriate, we need more personnel in the long run since the HIV clinic is heavily supported by its partners and is over loaded given the number of clients it has, so, if one day the partners walked away it means the people who should step in their shoes are the Doctors and Clinicians, so what is the minimum number of Doctors, Clinicians, nurses and counsellors to work in such a heavy loaded area ,therefore to me this is an area that really needs a lot of personnel to work in and also in its data section, so, if it is well organised, yes it can be started now. *(DR. OMALE ARRH)* - It will be good since the issue of adherence to treatment is managed and in terms of co-infection and human resource it can be achieved, so, integration will benefit both parties. *(Sr Susan ARRH)* - It is all about sensitization, if they get to know why sensitization is done it will be okay. When a patient comes to the hospital for service, he/she needs a lot and the first thing is introducing to them changes that are going around, convincing and counselling them, but when we don’t communicate to them effectively, with the culture and belief around them, they can easily be discouraged and they may not believe in what we are doing and run away. Therefore, with enough sensitisation, the clients will be comfortable with the integration. *(MARY Laboratory ARRH)* - It will be appropriate especially for the co-infected he/she can get all the services in the day. The patient who is co-infected is going to interact with more people unlike being always just with the same people in the clinic. *(HOSPITAL DIRECTOR ARRH)* - It is appropriate in the sense that hepatitis and HIV clients you will see the drugs these people use like new clocitis and new clotitis these are drugs that can be offered to these clients now in the same place not having the contradiction of referring a patient to the hepatitis clinic for a different prescription. Secondly, for hepatitis when a client is not handled well with the drugs, client develops resistance not even exceeding two years somebody has already developed drug resistance, therefore, integration will make the person to be managed in a good way. I think it is very important for our community to be sensitized because hepatitis alone is more infectious than HIV in terms of the public aspect. Therefore, offering these same services in the same place in this hospital we shall still need more sensitization because before the clinics were run separately, so obviously, when you want to bring change information has to be given to the community first otherwise people will have misconceptions that will discourage the clients from accessing the services. This system will be comfortable depending on what we shall do first that is ensuring that all the staff required for managing both HIV and HBV are empowered through training and have the necessary knowledge and skills and a set system in place. Then if we make sure there is a room where it can be run, then logistically if all those things are going to be in place and are very organised the integration will really run. *(ISAAC clinician ARRH)* - It will be appropriate because we also have other services we have integrated in to the HIV care like Family Planning and also they benefit from the Health Education which is given here because it will create awareness for them about the two diseases. I think the comfort will be built slowly and it all comes from the heath education that is given to them here to let them understand that these two diseases are somewhat related. (*Teddy HIV clinic data ARRH)* |
| **SATISFACTION** | Satisfaction with the integrated HIV/HBV care system | - If it comes on board I will be satisfied. May, be expansion of knowledge since we are going to have two things as a clinic. *(Dr. Wilson HIV clinic ARRH)* - If tested for HIV and Hepatitis, I would have known my status and it will help me to know whether to be on treatment or to be observed, proper diet, counselled or on health education. *(ADIRU NURSE ARRH)* - I will be satisfied if at all am handling a mother who has both Hepatitis and HIV and I want the mother to deliver the baby completely negative of the two diseases hence this will enable me to start monitoring her right from the beginning up to the last stage with taking more precautions to avoid transmission of mother to child. *(ADIRU NURSE ARRH)* - I will say 90% for integration because it will help me save the baby because I would have known the status of the mother before she delivers. *(ADIRU NURSE ARRH)* - I will be satisfied 70% with the integration reason being, as you start something new, the start of a program you cannot be sure with the success of the program until implementation starts and you gain experience with time. So, there may be those who will be doubting and resisting, but with time, perfection and education it will be appreciated. *(ADMINISTRATOR ARRH)* - I am very satisfied, in fact we the lesson we have learnt from the HIV clinic, we possibly use them to improve on hepatitis services, HIV for now has a very organised system and integrating the two means we are going to use HIV services as an example to build up on the hepatitis services and so I strongly agree that the integration will work. *(Clinician Koboko hospital)* - I will be satisfied on condition that the staff here should be trained, they must all have knowledge on Hepatitis such that at any corner they can be able to handle any hepatitis client and also the same should be done to the staff from the hepatitis clinic in order for them to have basic knowledge on HIV care and management. *(INCHARGE HIV CLINIC ARRH)* - I support this integration at 90%, simply because we are going to share resources which otherwise nobody thought about, because this is amidst challenges especially attitude, because those in the HIV and Hepatitis clinics were used to their setting only and did not want to learn anything and were purely employed to work on HIV and Hepatitis only and therefore, sharing of knowledge will be there for the two sides of the clinics if integrated *(DR. OMALE ARRH)* - Yes, I am satisfied with the integrated service and supported it with 70% because we had discussed the benefits when it was managed different and if integrated it will move on well. *(Sr Susan ARRH)* - I will be satisfied with 80%, because when i usually go to these two clinics and see how they are doing, I see if there is a possibility of merging them together definitely, they will be happy and even the community at large will appreciate. *(MARY Laboratory ARRH)* - As of now the merge is not yet there, however, I will be satisfied up to 95%because i will not have to look for another room for hepatitis since they are going to under one roof. *(HOSPITAL DIRECTOR ARRH)* - To a larger extend i will be satisfied with the integration only if what is required is put in place, because this is not the first integration the ministry of health is bringing in place, other integrations like HIV and TB have worked and are already working very well, so what the government needs to do is to make all it takes for this integration to work is very available, so I will give it 60% to work. *(ISAAC clinician ARRH)* - I cannot rate the satisfaction because it has not yet began. Now here we have issues with follow up of clients in TB, HIV Clinic and PMCT and data as well. In terms of percentage, I will give it at 70% out of 100% because here I am looking at a real time data. Therefore, I am looking at the data flow since all these have stages of data capturing and Ministry of Health also have its own registers which have to be filled and I am now looking at it if someone wants real time data maybe for a weekly report, like how many came, missed and how many are due. Sometimes it is not easy to get all this data and now in my head I am looking like all this is going to be much work added like a certain number of Hepatitis clients and sometimes this may improve if there is an increment in the man power to put it right. (*Teddy HIV clinic data ARRH)* |
|  | Dissatisfaction with the integrated HIV/HBV care system | - Quality of service minimized due to increase in workload, service delivery will be tiresome especially for health care workers of which they will need some allowances and salary increment. *(Dr. Wilson HIV clinic ARRH)* - The 10% is for those who come in the rush hours without knowing their status but need to be helped first. *(ADIRU NURSE ARRH)* - Secondly, putting myself in the shoes of the in charge of ART clinic, starting a new thing is always hard and at times scares and the number of patients going to be seen is going to increase and also there is need to change the way of doing things in the clinic for instance, need to get more furniture for the increasing number of the patients ,and all these at the beginning are all going to be worrying, however, with time and change of attitude and getting used to the work things will normalise. *(ADIRU NURSE ARRH)* - Generally, the 30% is for the initial phase of the integration since there will be many people doubting the success of the integration and also the provider and receiver interference because the two will be having one line of service flow. *(ADMINISTRATOR ARRH)* - I will not be satisfied if all the requirements needed for this integration to be possible are not put in place especially the issue of human resource, logistics and even space since the HIV clinic is already congested with the current population seeking services in the clinic. *(INCHARGE HIV CLINIC ARRH)* - The 10% is for uncertainties along the way of integration and also, I cannot be sure of what will come with the integration because it has not yet started and also the approach used to bring it in form of a project makes me to doubt its sustainability. *(DR. OMALE ARRH)* - I am not satisfied with 30% because of challenges of having big number of clients going to be seen by the limited number of staff which will lead to poor quality service and control measures. It has not yet also started so its assurance is not yet determined and guaranteed. *(Sr Susan ARRH)* - I will not be satisfied with the integration with 20%, as I said early on, for us in the laboratory we may not cater for the patients due to equipment break downs and reagents stockouts hence failing to satisfy the needs of the clients however, if such happens in the course of the integration, the clinicians may not be happy with us and this is why i see it may not satisfy the integration. *(MARY Laboratory ARRH)* - I will not be satisfied with 5% because, where many people are in attendance for services, quality is always compromised and having specialities is always good for high quality services. *(HOSPITAL DIRECTOR ARRH)* - Percentage wise am not satisfied with 40% because you put HIV and HBV activities together and we don’t have medicines, essentials from the laboratory where these activities can run, we do not have a well-built system in place where at one level of management if we start the implementation there is no one we can consult and then no staff who have been empowered in terms of capacity training to manage this integration very well and then a system where everything is just being pushed to you whatever happens no one cares about it, therefore, at that level I will be dissatisfied. *(ISAAC clinician ARRH)* - If it is manual definitely, I will not be satisfied. Manual systems are not easy; it’s easy to do data manipulation if you have the system because with manual you are bound to miss many things. (*Teddy HIV clinic data ARRH)* |
|  | Community belief about the service | - If we handle things well there will be user friendliness. May be with the slight increment in the population of clients received it may lead to more HIV clients asking for transfer outs to nearby facilities and this is something I fore see. *(Dr. Wilson HIV clinic ARRH)* - The community may not be all that worried, but because of stigma, may be some may get lost, ask questions why the clinic has been taken to HIV and yet am just having Hepatitis but not HIV, so with time I think the attitude will change. Therefore, before the integration is started, the information has to be passed first to the population and the reason as to why we are integrating has to be passed clearly so that they will not ask a lot of questions, and once they come to the hospital they already know where to get their treatment. *(ADIRU NURSE ARRH)* - People will take the service simply because of its centrality, delivery will be in one place this will reduce time wastage in case you are to split and they will benefit from the expertise of the knowledgeable health worker because of the empowerment the health worker got through knowledge and skills training. *(ADMINISTRATOR ARRH)* - As for now we have not registered any complain from the health workers in terms of this integration may be probably because it has not yet started however, I see it is going to be user-friendly both to the clients and the health workers. *(Clinician Koboko hospital)* - They will not have much since they want service. When they are given health education they will understand and accept to come under one roof. For those infected it will be user friendly especially those who are sickly. *(INCHARGE HIV CLINIC ARRH)* - People in our community will take up the service because first of all they are already aware of the dangers of hepatitis coupled with the sensitization both in the clinic and outside, surely, they will have to. *(DR. OMALE ARRH)* - It will be user-friendly especially preference to the clients on whether to come for Hepatitis or HIV clinic for treatment. The administration will be saved in terms of the existing space and personnel is already there in the facility. *(Sr Susan ARRH)* - People will find it friendly, just as I had told you, if they are sensitized, they will find it easy because of the health education given to them in the clinic and the sensitization should not only stop at the clinic level even in the radio, churches and within the community through dialogues. *(MARY Laboratory ARRH)* - They will know there is a clinic that attends to all health problems. Therefore, the community will believe in the service. When a generic name is put, the perception of self-stigma will be reduced. *(HOSPITAL DIRECTOR ARRH)* - Currently the uptake of the integration is not good because we as ART clinic have not been put before this program starts. When everything is put in good place then people can easily change their mind to take on the integration. *(ISAAC clinician ARRH)* - In the ART CLINIC here the first thing we do when we arrive is offering health education to the clients, which takes like 2 to 3 hours sensitizing clients because we have staff who have been employed as health educators. So, currently the hepatitis clients do not understand into details what takes place here in the HIV clinic and the same happens to the HIV clients they are not aware of what is happening in the hepatitis clinic because the two are being run separately. *(ISAAC clinician ARRH)* - I won’t commend much on that because I do not know what is there in the package for us. For the health care workers as long as they are trained, they will be okay. As for us in data nothing is impossible as long as everything is streamlined. For the clients I think it will be user-friendly because they will be having extra services besides what they have really come for like prep, family planning and health education instead of coming for hepatitis alone. (*Teddy HIV clinic data ARRH)* |
|  | Views of people in community about satisfaction with HIV/HBV integrated care system | - Yes, they will be satisfied, if they are told the reason for the integration and its importance during health education sessions. *(Dr. Wilson HIV clinic ARRH)* - Some people will be satisfied and others will not this is simply because when you bring an idea in the community, not all will say yes and others will have their own reasons for saying NO and YES. Besides, there will still be some limited satisfaction somewhere amidst those who are satisfied. *(ADIRU NURSE ARRH)* - Patients will be more satisfied because of the package. The health workers will also be satisfied given time, when all their fears and concerns are being dealt with through the appropriate mechanisms, training, sensitization, continuing medical education when the integration takes off. *(ADMINISTRATOR ARRH)* - Our staff are willing to take up the integration and I think what they need for now is only training and knowledge for this and currently the staff are picking it positively. *(Clinician Koboko hospital)* - Stigma is always there however much they are sensitized especially the hepatitis clients will complain why they have been transferred from their clinic to the HIV clinic and yet they are not HIV positive coupled with the issue of being spotted by friends and relatives in this clinic so stigma is going to be prevalent. *(INCHARGE HIV CLINIC ARRH)* - Concerning the views of the people about satisfaction with this service, I personally cannot now determine that because people have different views ranging from patients to health workers, however, for health workers am seeing it from the work load aspect where they are going to complain of being over worked. *(DR. OMALE ARRH)* - From the client’s perspective, especially the hepatitis patients fear to take their medication from the HIV clinic simply because they are not HIV positive and also fear being seen taking treatment from that clinic by their friends. *(DR. OMALE ARRH)* - Community will be satisfied with the integration because the notion in west Nile is now that you better get HIV than hepatitis and once it is integrated, they will get what they want and will be satisfied. *(Sr Susan ARRH)* - You see the people in our community I don’t think they are still ignorant, people are already educated, from the time we started screening people for hepatitis here in the hospital and also in the community, people got sensitized so they are already highly educated about Hepatitis and HIV and from the district there are teams and also in the villages, teams are there especially the VHTs, so all these teams are well educated and once you take that line they really understand that Hepatitis is a dangerous viral disease like HIV, so when there is that integration they will be aware. *(MARY Laboratory ARRH)* - I think the only view of the health workers will be how the clinic will be called after the integration otherwise, for the clients they will be satisfied since they are going to receive a comprehensive care service. Secondly with the continuous sensitization and health education, people will realise the importance of the integration and gradually will be satisfied with the services delivered. *(HOSPITAL DIRECTOR ARRH)* - In the community I have not really engaged myself to determine the views of the people unless we do a survey we would come up with their views. However, for the health workers as of now their views are still negative one is because of the staff number which is not enough to manage the clients and yet the number of patients seen by the few is so big and their fear is that if the hepatitis clients are added on top of the existing ART clients there is going to be extra work and space is also not enough and for this extra work are they going to be facilitated or another clinic day is going to be set for hepatitis. *(ISAAC clinician ARRH)* - Everyone has his/her own perspective of this integration like how it will be and work, for instance people in the file room will be saying will the file room not be congested with a lot of work meanwhile those in the triage will say are they going to have different clinic days for HIV and HBV so majority of the workers have mixed ideas because all of them are not aware of what is in the package. For me personally, am looking at a scenario where they are giving me more workload like the hepatitis files and that alone just scare me. Therefore, the satisfaction aspect of it cannot be predicted since it has not yet commenced as we are still eagerly for its start to see what will happen. (*Teddy HIV clinic data ARRH)* |
| **INTENTION TO USE SERVICE** | Willingness to use Integrated care service | - I am willing to take on the system if some adjustments are made in the system because we have workers at every point of care ranging from testing, laboratory, counselling and pharmacy. I know hepatitis clinic also have their own records, so what do we do with the records. Are we going to have one different clinic days for HIV and HBV is what needs to be adjusted. *(Dr. Wilson HIV clinic ARRH)* - I am willing to use the services and it is not going to be a new thing to us only that we shall have to be consistent. *(ADIRU NURSE ARRH)* - Personally, I am even ready to use the service given the advantages am seeing it is coming along side with. *(ADMINISTRATOR ARRH)* - You see sometimes new innovations are accepted or rejected depending on the mode of introduction. The introduction of the integration should be in a systematic and consistent way with empowering the health workers first making them to understand the whole thing, this will strengthen them to make sure they are ready for the task ahead of them and then it will attract commitment to embrace it and once that is done, for the client it will just be obvious. But if it is just being thrown to the health workers just like that without them being embraced coupled with the fears I said earlier, it might pause a problem to use the integrated service. Otherwise, we are ready for it is being introduced the way I had mentioned before. *(ADMINISTRATOR ARRH)* - I am willing based on the fact that I see it beneficial to the clients because it is built on the package content of the clients, something that is going to build up the knowledge base of our health workers and capacity of our staff and something that will build up our data system hence having a new timely data management. *(Clinician Koboko hospital)* - Because it is a government policy I will not sabotage let’s give it the chance because we can use it to improve our discipline and there may be good things and therefore, I am ready to use it. *(INCHARGE HIV CLINIC ARRH)* - I am willing to use the integrated service simply because of the challenges of running isolated clinics given the few resources versus the resources we have is what increases my interest of having integration. *(DR. OMALE ARRH)* - I will be willing to use it because we have ever integrated other services and are now working well hence, I do support this move. *(Sr Susan ARRH)* - We are willing to use it, in fact for us here we are already integrated and just ready to embrace the integration. *(MARY Laboratory ARRH)* - People will not resist, especially those with both hepatitis and HIV will not say that they want to be in their own facility. *(HOSPITAL DIRECTOR ARRH)* - Am willing to use this service because the government comes with its policies and we who are down here have to implement what the government has come up with because i believe government cannot come up with bad interventions because they want the country to have a healthy population. *(ISAAC clinician ARRH)* - I am much willing to use it at 100% as long as my fears are catered for like having more registers, increasing the number of personnel and increasing the space for file room obviously everything will be in line. (*Teddy HIV clinic data ARRH)* |
|  | Intention to use the integrated care service | - Yes, I am ready to use the integrated system. *(Dr. Wilson HIV clinic ARRH)* - Definitely we need to use this integrated care system. *(ADIRU NURSE ARRH)* - Yes, people will use the integrated service. *(ADMINISTRATOR ARRH)* - We are so much willing to take up on this integration as soon it is rolled on to us and see how we can utilise the little available resources. *(Clinician Koboko hospital)* - We are going to use it in fact we have already started this integration because here we have both TB and HIV treatment and we want to embrace it all. *(INCHARGE HIV CLINIC ARRH)* - As workers we may not have a lot of resistance, however, the challenge to me from the workers side is the knowledge gap, so, before we start the integration, there should be a pre orientation for either side for what they are supposed to do on these clients instead of someone presenting himself there and do not know what he is supposed to do. *(DR. OMALE ARRH)* - We want to use this integrated service and see its viability. *(Sr Susan ARRH)* - Integration is the only thing we want to showcase as I had told you for us here in the laboratory we have already integrated. *(MARY Laboratory ARRH)* - We intend to use the integrated service because it is a good move. *(HOSPITAL DIRECTOR ARRH)* - I intent to use it when all what we are demanding is availed to us. *(ISAAC clinician ARRH)* - I am ready to use it, if the marriage is supported very well. (*Teddy HIV clinic data ARRH)* |
|  | Whether Integrated HIV/HBV care service will be used by patients / clients seeking Hepatitis B care services | - It will be taken up by the clients because if one has both HIV and HBV probably in the long run it will reduce the stigma. *(Dr. Wilson HIV clinic ARRH)* - If they are well educated in the beginning since it is a policy from the Government, they will take it up and will have NO choice to refuse it. May be the only challenge may be lack of space because the current space in the HIV clinic will not really be enough to accommodate all that population. *(ADIRU NURSE ARRH)* - Yes, people will use the integrated service if once it is introduced in a very systematic and consistent manner, even the hepatitis clients will be in position to use it well. *(ADMINISTRATOR ARRH)* - I think they will be so much willing to take the integrated care service. *(Clinician Koboko hospital)* - Yes, believe when these hepatitis clients are given the opportunity to understand the reason of this integration and the importance that is going to result from it and with continuous sensitization of the people about these services they will come willingly for their treatment since our district and the region at large has a high prevalence rate of hepatitis which has scared so many people and they are all eager to be treated for. *(INCHARGE HIV CLINIC ARRH)* - The patients will be ready to use the integrated service after telling them the reason and importance of the integration. *(DR. OMALE ARRH)* - I do not think patients will refuse its use because they are already on treatment and it will instead even benefit those who are co-infected more since they will receive all their medicines under one roof. *(Sr Susan ARRH)* - They will be ready to use the integrated care service because they always do whatever the health workers tell them to do and I don’t think they will refuse as long as they understand the good of the integration, they will be ready to use it. *(MARY Laboratory ARRH)* - The integrated service will be used, as we merge, we even expect the numbers to rise with availability of space, consultation rooms and waiting shades for patients. *(HOSPITAL DIRECTOR ARRH)* - What we do is when we discover a client is on hepatitis B care, we make sure these clients are initiated on TDF-3TC regimen in case they do not have any renal contra-indications, so for those we have been seeing, we change them to TDF base regimen after doing liver function test. So, for those follow-ups like liver function test and viral load tests, we have been referring them to the physician that is Dr Omale. Therefore, we have actually been on the integration but not at the higher level. It will be useful to the patients when we give proper health care education, sensitization over the radio and also involving other stakeholders like politicians in the campaign. I will say they will walk to the facilities because no one has gone to the community to pull any one to come for treatment because these people are already on care in the hospital, so it just the matter of joining the two clinics together and the care continues. *(ISAAC clinician ARRH)* - I believe if these people are told the importance of the integration during the health education sessions how the two diseases are going to be managed in a gradual process, they will be aware of what they are going to receive, they will come for the services. (*Teddy HIV clinic data ARRH)* |
|  | Recommendation for people in the community to use the Integrated HIV/HBV care service | - I think for now I will recommend for district hospitals but not for lower health facilities since they do not have other systems like those we have ever tried to visit. *(Dr. Wilson HIV clinic ARRH)* - I will recommend the integrated care service for excellent service delivery. *(ADIRU NURSE ARRH)* - There is benefit from the experience of the health worker, the empowerment training which the health worker will have, therefore, it will benefit all the clients across the flow, therefore, I will recommend it to be used by the people. *(ADMINISTRATOR ARRH)* - Yes, I will really recommend people to take up this integrated service because it has a lot of good things ranging from the variety of services offered to even its convenience to clients who are co-infected. *(Clinician Koboko hospital)* - I will recommend people to use this integrated care service. *(INCHARGE HIV CLINIC ARRH)* - I will recommend this integrated service because it comes with multiple advantages. *(DR. OMALE ARRH)* - Well, I do recommend the integrated care service. *(MARY Laboratory ARRH)* - I do recommend the integrated care service as for me I see it now the only way to go for better service delivery. *(HOSPITAL DIRECTOR ARRH)* - Yes, I will recommend, my reason will just be for a holistic management of a client who has both conditions. *(ISAAC clinician ARRH)* - We need to see what happens and see whether it can work first before I commend. (*Teddy HIV clinic data ARRH)* |
|  | Readiness to change to Integrated care service | - As for care providers I think they are very ready because we have a different clinic day for children and therefore, we shall have to reorganize the days especially for HBV and HIV. *(Dr. Wilson HIV clinic ARRH)* - We are very ready to change because many of our mothers have missed that opportunity, we do not check them for Hepatitis and yet some come when they are hepatitis positive, however, it has just been the initiative of the mothers to go and test willingly and come back to us with the results. Therefore, if it is going to be integrated, it is going to save the lives of very many babies through paying keen attention and testing these mothers first. *(ADIRU NURSE ARRH)* - We are very ready to change, because as of now i think integration is the only way to go because we suffering from staff crisis here in the hospital. . *(ADMINISTRATOR ARRH)* - we are ready and shall need a few things like issues to do with data management for hepatitis and training for health workers and also a facility based training on the management of hepatitis and HIV, sensitization of the health workers that we going to be having hepatitis and HIV services run together in the HIV clinic for some defined reasons and also creating awareness among the HIV clients that we are going to have the hepatitis clients here taking their treatment together with them in the clinic so that they can understand that the resources are meant for all of them such that they don’t harass each other. *(Clinician Koboko hospital)* - I am personally ready for this integration because it is not the first integration here and what I want is only to see every that is needed should be in place such that we make this integration to be part of us since it is providing a holistic approach in addressing certain gaps in our hospital setting and improves the management of several illnesses under one roof and by few clinicians and nurses. *(INCHARGE HIV CLINIC ARRH)* - We are very ready for this integrated service because this region is having high population of people with Hepatitis as I had told you before we have screened so many patients here in this hospital for this disease and actually, we want to see it start quick such that our people can start on the treatment. *(DR. OMALE ARRH)* - I will recommend this integrated care service because we already have structures in place like we do follow ups and reviews. *(Sr Susan ARRH)* - Within the laboratory its already existing and we are working under the same roof, all samples come here and we analyse them here. *(MARY Laboratory ARRH)* - This should be the way to go and where we get partners because the interests have always been parallel, now this should take one direction such that we just have to go with the integrated model. *(HOSPITAL DIRECTOR ARRH)* - I would be ready but since this is a regional hospital, above me there are so many people, so if the ministry has engaged people above me and they give us the permission, we shall obviously take on the integration. You know you might start an activity but you might not sustain it, that is why throughout this session you have been asking me about the suitability of this program and ow you want the sustainability. That is why before i said we must not be on hurry to start something. So, I believe by the time the Government has gone up to this extent they should be ready to sustain the whole process, so sustaining will need lot for the whole proves and all what we need to be putting in place is all that we required during the board meeting. So, if all what we required has all being solved, then everything will be sustainable. *(ISAAC clinician ARRH)* - Readiness has many factors to say that am ready, if say all those factors I mentioned early on are in place my fears are addressed. (*Teddy HIV clinic data ARRH)* |
|  | Likelihood that the integrated service will be sustained? | - For sustainability, I think if all those systems I mentioned in the beginning are put in place this integration will be sustainable. *(Dr. Wilson HIV clinic ARRH)* - It will be sustainable as long as the combined knowledge are there and then the equipment that are supposed to be used are there, it will be taken up. *(ADIRU NURSE ARRH)* - I think it will be sustainable however, it will also need to come with a lot of support such that it can be sustainable because there are certain gaps that needs to be bridged in terms of the human resource. I see a scenario where the concern of human resource is going to come, while it is better have the integrated service because you may not need more human resource but the increase in the scope and range of services within our hospital here will still call for more human resource. *(ADMINISTRATOR ARRH)* - Also, in terms of medicine and supplies, there may also be need because of the shortages which we go through, just across the board it may not just be only those areas which we are discussing. *(ADMINISTRATOR ARRH)* - Sustainability can also be supported by promoting human resource and strengthening human resource capacity especially those who are already there. Human resource has to be empowered through appropriate training and transfer of knowledge. *(ADMINISTRATOR ARRH)* - I believe if the system is established in a way that enough sensitization is done for all people in the community ranging from the health workers to patients and the public at large, obviously after two five months on road people will know where the integrated care service is being offered here in the hospital and will even know where to go for these treatments and they will embrace it hence it will be sustainable. *(Clinician Koboko hospital)* - This integration will be sustainable if all the stake holders have their input in it and therefore, they will all aim at seeing it reaches its full potential by availing everything that is needed to ensure that it is effective and consistent such that we don’t lose our clients on the way since this is a lifelong treatment plan. *(INCHARGE HIV CLINIC ARRH)* - The first sustainability plan is the involvement from the hospital top management and to managers of the clinics especially HIV, where partners are also part of the management so that they own this and putting mechanisms to see staff rotation and deployment in the various departments. Once, these are done it will be sustainable. *(DR. OMALE ARRH)* - May be the only resistance I see for workers is only the workload, so, personally am ready for this integration, so, you know people do not want to embrace change at the beginning but with time they will believe it. *(Sr Susan ARRH)* - Concerning sustainability, as I had said before we have ever integrated other services therefore, it will have to be sustainable. *(Sr Susan ARRH)* - In the laboratory, man power is not a problem, we just have to re-organise ourselves and I had told you we are already working on the patients, may be our problem may arise from the shortage of supplies. *(MARY Laboratory ARRH)* - When we bring it in a project model to me it will not be good, instead we should be able to introduce it as a service re-arrangement so that it fits within the main stream. When in project mode it will need additional resources to run it because projects have time frame so you will see sustainability problems arising ranging from compensating staff time and stocking medicines which will in the long run require you to put in more personal resources to sustain it. *(HOSPITAL DIRECTOR ARRH)* - Integrating TB and Family planning is not a chronic care, Hepatitis is where we need to be more prepared than we are prepared for TB and Family planning because the care is also going to be like HIV care, once you have made a diagnosis of chronic hepatitis B this is a lifelong care, so there we have to be more prepared and the process of sustainability should be continuous. *(ISAAC clinician ARRH)* - The sustainability view point of it, I have not given it much thought yet because it requires constant intervention and reviews. (*Teddy HIV clinic data ARRH)* |
|  | Additional information included in the discussion | - May be additional support the integration will give the hospital. It may be feasible of course when you are starting something new there are a lot of things to be put in place like logistical support like the hepatitis clinic will be having challenges *(Dr. Wilson HIV clinic ARRH)* - Actually, we have discussed everything, however, we need skilled personnel to disseminate the information to the population in order to make it move on well. *(ADIRU NURSE ARRH)* - Another most important thing is to train the health workers on what is to be done first so that they can handle the mothers and community very well. *(ADIRU NURSE ARRH)* - I just want to emphasize on the service providers/health workers fears about the integration concerning work over and this has to be rectified such that the focus has to be set clearly on service delivery to ensure that the clients receive the services adequately. *(ADMINISTRATOR ARRH)* - Possibly I would like to know how long you intent to roll this integration out and when it is going to start and also whether some specific days are also going to be set for seeing the hepatitis clients or they will also be managed throughout the course of the week. *(Clinician Koboko hospital)* - I would like to see all the stake holders in this cause to fully cooperate with the health care workers and support each other where necessary starting from the hospital administration up to the clinic to ensure that our people receive treatment effectively because we are here being entrusted by the ministry of health to ensure that we have a healthy nation that is being served by professionals. *(INCHARGE HIV CLINIC ARRH)* - May be what I can add is about the partner support that it should be continuous without breakages because once this is withdrawn it will affect the integration especially for the case of partners that are being funded therefore, there should be another sustainability plan set aside in case one option fails the other can be applied to maintain consistency in service delivery. *(DR. OMALE ARRH)* - You in Uganda, resources will not be enough, I have been here in the hospital for the last fifteen years and it has expanded as a referral hospital however, staffing has been our only challenge here but now the population in the region has grown which also necessitates more structures to be put in place to meet the growing population each time and other issues will work out but the case of human resource is biting. I would also advice that we move on a slow pace kind of giving it a trial and if we see it moving smoothly, we shall expand. But it is also better for it to be given time like the way a plane gets its momentum to take off such that the people can embrace it in the long run. I would also advice that our physician should be one of the most key informants in this exercise because he already knows what it takes since he has had the worst experiences in that area, if you had told me that you had interviewed him, I would not have accepted you to interview me because he has fought too much for this Hepatitis programme. *(Sr Susan ARRH)* - Maybe I would only urge the staff from the two clinics to come together and work as a team for better results. Secondly, if they can train some peers in the hepatitis clinic like those in the HIV clinic to talk to their fellows, I think it will work. *(MARY Laboratory ARRH)* - The issue of resources since this is a project and usually projects have a life span how shall we be in position to push further with this integration once the project period expires so if possible the implementing partner together with the hospital administration should be in position to lobby for some funding from interested funders to help us to continue with the integrated service because for me i see it is something that has already worked for us though we have not yet started the implementation process. *(HOSPITAL DIRECTOR ARRH)* - What I would like to know is the issue of viral load, how viral load will be done for this clients, secondly, the issue of scanning machines and issue of chemistry in the laboratory, most times the machine is off and on, so those key things and the tests we normally do for hepatitis B clients, how far have you people gone as far as the availability of those things are concerned. *(ISAAC clinician ARRH)* - We do follow- up for the non-suppressed clients, lost clients and those coming for the second visit, and I do not know how you are going to do it that is why I said I do not know how you have prepared it in your package. *(Teddy HIV clinic data ARRH)* - There is need for more eye opening on hepatitis since people have scanty information about it. If you talk to people, they will give you their own views as they are having limited information about it, but if we broaden the dissemination of the information about hepatitis people will embrace it. *(Teddy HIV clinic data ARRH)* - Lastly, I want to know whether people/healthcare workers will be remunerated for this extra work. *(Teddy HIV clinic data ARRH)* |
